# Supplementary material for: A meta-epidemiological assessment of transparency indicators of infectious disease models
Source: PLoS One. 2022 Oct 7;17(10):e0275380. doi: 10.1371/journal.pone.0275380 (PMC9543956; doi:10.1371/journal.pone.0275380)
Supplement: Attachment — Submitted filename: Response to Reviewers.docx [file pone.0275380.s001.docx]

July 9, 2022

Chi-Hua Chen, Ph.D.

Academic Editor

PLOS ONE

Re: PONE-D-22-14026

A meta-epidemiological assessment of transparency indicators of infectious disease models

Dear Dr. Chen,

We were pleased to hear that PLoS ONE is interested in a revised version of our paper. We thank the editors and the external reviewer for the helpful comments. We have addressed these comments in the current revision. In detail:

*Editorial/formatting/ formal matters:*

The statement for competing interests has been adjusted (thank you for changing it in the submission form) as you proposed in the letter. Furthermore, the formatting has been adjusted so that it follows the journal requirements and standards and we have tracked all changes in a document so you can follow what has been changed. Finally, the code and data availability statement still holds, and, in our references, there are no retractions to our knowledge and after surveying the list we find no such.

*Reviewer 1 comments:*

Reviewer #1: This is a very interesting and thought provoking article. It is a common sentiment that scientific rigour and due process has been sacrificed during the course of the pandemic over a perceived notion of urgency, or sometimes over the need of beating others to the punch and claiming the glory. Not surprisingly, mathematical models of infectious diseases became darlings of the main steam media due to their striking, often sensational, conclusion which were very suitable as headlines. More importantly, they were often used to guide public health policies that had impact and will have lasting consequences on the society. I congratulate Dr. Ioannidis and Zavalis on their work and my opinion of the paper is positive.

Reply: We thank you for the kind consideration of our work and for the positive appraisal.

The search strategy appears to be robust and they opted to screen the retrieved citations with a single author. This is rather unconventional but unlikely to change the outcomes but it would be worthwhile to explain why they opted to do so.

Reply: We admit it may be a bit unconventional to only have one author (EAZ) screen the eligibility, but we proceeded with a single author screening the papers essentially two times as papers were also assessed during extraction of disease modelled and model type. During the first screening round the exclusion was more unrestricted/inclusive and in unclear cases they were included to be judged more stringently when EAZ read the full article to extract disease modelled and model type. We presumed that it wouldn’t affect the results in any dramatic way and would help streamline our approach to the data. After all the paper either contained a model or no model and we considered the definition included in our methods stringent enough to not allow for too many degrees of freedom in the inclusion of articles. We have added in the limitations: “Using only one main reviewer for screening for eligibility may have introduced some errors in the selection of specific studies that were included or not in our analysis. However, identification of eligible studies is quite straightforward given our eligibility criteria and any ambiguous cases were also discussed with the second author. There were a few studies that did not fit squarely in our pre-determined categories, but their number is too small to affect the overall results.”

Using an R package to extract the information is wise and likely to reduce any inadvertent mistakes. Verification of the automated extraction with random checks is also good practice. I would have personally opted to verify any positive findings for variables in which the algorithms showed less than 95% PPV but complete random checks are also fine and a different methodology is unlikely to change the conclusions.

Reply; We agree.

Findings are self-explanatory and unfortunately, quite damning. It is inconceivable that a mathematical model would be published without open access codes, and I would not mind if a stronger language was used to criticise these points. While Dr. Ioannidis stated pre-registration may be cumbersome, I believe it is entirely feasible and several items (assumptions, data sources to be used, general structure of the model...) can and should be made public before modeling begins so readers can appreciate the evolution of the algorithm whether it was natural or forced to achieve a specific end.

A mathematical model SHOULD NOT be taken seriously, talked about or published WITHOUT the underlying code and data sources are made public. This includes the models published by academic journals or unpublished ones that were used to guide health policies alike.

Reply: We certainly agree with the reviewer’s comments pertaining to the importance of transparency in such an influential field and how it has been neglected during the pandemic with differing excuses. We have added some stronger language along the lines of what the reviewer proposed: “It can be argued that a mathematical model should not be taken seriously, especially for influential inferences and decisions, without the underlying code and data sources made public. This includes models published by academic journals or unpublished ones that are being used nevertheless to guide health policies or other decisions. One might even suggest banning the publication of models that do not share their data and code. Pre-registration also is highly desirable, whenever pertinent, and for some targeted uses of models, e.g. making claims for future predictions, it should become the normal expectation.”

We thank you again for the constructive comments that have helped us improve our work. Having made these revisions, we hope our work would be acceptable for publication in the current revised version

Sincerely,

John P.A. Ioannidis, MD, DSc

Professor of Medicine, of Epidemiology and Population Health, and (by courtesy) of Biomedical Data Science, and of Statistics

Co-Director, Meta-Research Innovation Center at Stanford (METRICS)

Stanford University
