# Supplementary material for: A meta-epidemiological assessment of transparency indicators of infectious disease models
Source: PLoS One. 2022 Oct 7;17(10):e0275380. doi: 10.1371/journal.pone.0275380 (PMC9543956; doi:10.1371/journal.pone.0275380)
Supplement: Attachment — Submitted filename: Response to Reviewers.docx [file pone.0275380.s002.docx]

August 28, 2022

Chi-Hua Chen, Ph.D.

Academic Editor

PLOS ONE

Re: PONE-D-22-14026

A meta-epidemiological assessment of transparency indicators of infectious disease models

Dear Dr. Chen,

We were pleased to hear that PLoS ONE is interested in a revised version of our paper. We thank the editors and the additional external reviewer for the helpful comments. We have addressed these comments in the current revision. In detail:

*Reviewer 2 comments:*

Reviewer #2: The manual validation process should include some negative examples as well as positive examples. The authors claim that this is unnecessary due to the transparency indicator extraction algorithm's high NPV in prior work. However, the PPV in this study was substantially lower than in their previous paper. The authors rightly acknowledge that this body of research is substantively different from the literature with which the algorithm was developed. Moreover, the conclusions of the paper largely rest on the shockingly low frequencies of many transparency indicators. Without manual review of negatives, however, it is uncertain how frequent false negatives may be.

Reply: We thank you for your input and we have since validated the negatives and added that information to the manuscript in the Results, and we have also added information accordingly in the Methods, and in the Abstract and modified the Discussion of the paper accordingly.

The authors do not address the multiple comparisons problem in their statistical tests. While it seems unlikely that it would affect the results greatly, this study design is certainly subject to this problem. The authors should either adjust for multiple comparisons or defend their decision not to do so.

Reply: As for the multiplicity all the statistically significant results in our paper have very low p-values that would survive any stringent multiplicity correction. We have added that “Statistical significance was claimed for p<0.005 and p-values between 0.005 and 0.05 are considered suggestive, as previously proposed”.

The regression analysis is not adequately described. In methods, the authors state "we would conduct multivariable logistic regressions as well", without describing any of the details of these models (IVs, DVs, etc). In results, the authors state "Multivariable regressions (not shown) showed similar results." Similar in what sense? Including these results could be more convincing than the Fisher's exact tests since they may be less affected by the multiple comparisons problem. (This, of course, depends on the details of the analysis, which are not clear as written.)

Reply: We thank the reviewer for their comment; we have edited the description of the regression accordingly.

In limitations, the authors mention that the sample of PMC papers is not representative of all PubMed indexed papers. Much of the covid modeling literature has been published as preprints or in other non-indexed sources. It would be interesting to compare covid research published in such venues to peer-reviewed covid research. Worth mentioning in the limitations and perhaps a promising follow-up to this paper.

Reply: An interesting thought indeed. Some of the papers in our sample were pre-prints as some pre-prints are indexed in PubMed, but most are not. We have added our thoughts on the pre-prints and the non-indexed literature.

The expectation of pre-registration when using models to make future predictions seems unrealistic. It is unclear to me how this would work. Even in such cases, modelers often don't know the final form the model will take when development begins.

Reply: As we discuss in the paper, we understand that the registration of models is not suitable for all types of models but for such that would inform policy we see registering the models as both a feasible and ethical thing to do.

We thank you again for the constructive comments that have helped us improve our work. Having made these revisions, we hope our work would be acceptable for publication in the current revised version

Sincerely,

John P.A. Ioannidis, MD, DSc

Professor of Medicine, of Epidemiology and Population Health, and (by courtesy) of Biomedical Data Science, and of Statistics

Co-Director, Meta-Research Innovation Center at Stanford (METRICS)

Stanford University
